# Downstream signaling and genome-wide regulatory effects of PTK7 pseudokinase and its proteolytic fragments in cancer cells

**DOI:** 10.1186/1478-811X-12-15

**Published:** 2014-03-11

**Authors:** Vladislav S Golubkov, Alex Y Strongin

**Affiliations:** 1 Cancer Research Center, Sanford-Burnham Institute for Medical Research, La Jolla, CA 92037, USA

**Keywords:** Protein tyrosine kinase 7, PTK7, Cell migration, Cadherin-11, Metalloprotease, CREB, ATF1

## Abstract

**Background:**

The full-length membrane protein tyrosine kinase 7 (PTK7) pseudokinase, an important component of the planar cell polarity and the Wnt canonical and non-canonical pathways, is a subject of step-wise proteolysis in cells and tissues. The proteolysis of PTK7 involves membrane type-matrix metalloproteinase (MT1-MMP), members of the Disintegrin Domain and Metalloproteinase (ADAM) family, and γ-secretase. This multi-step proteolysis results in the generation of the digest fragments of PTK7. These fragments may be either liberated into the extracellular milieu or retained on the plasma membrane or released into the cytoplasm and then transported into the nucleus.

**Results:**

We employed the genome-wide transcriptional and kinome array analyses to determine the role of the full-length membrane PTK7 and its proteolytic fragments in the downstream regulatory mechanisms, with an emphasis on the cell migration-related genes and proteins. Using fibrosarcoma HT1080 cells stably expressing PTK7 and its mutant and truncated species, the structure of which corresponded to the major PTK7 digest fragments, we demonstrated that the full-length membrane 1–1070 PTK7, the N-terminal 1–694 soluble ectodomain fragment, and the C-terminal 622–1070 and 726–1070 fragments differentially regulate multiple genes and signaling pathways in our highly invasive cancer cell model. Immunoblotting of the selected proteins were used to validate the results of our high throughput assays.

**Conclusions:**

Our results suggest that PTK7 levels need to be tightly controlled to enable migration and that the anti-migratory effect of the full-length membrane PTK7 is linked to the down-regulation of multiple migration-related genes and to the activation of the Akt and c-Jun pathway. In turn, the C-terminal fragments of PTK7 act predominantly *via* the RAS-ERK and CREB/ATF1 pathway and through the up-regulation of cadherin-11. In general, our data correlate well with the distinct functionality of the full-length receptor tyrosine kinases and their respective intracellular domain (ICD) proteolytic fragments.

## Background

Pseudokinase PTK7 is a Wnt co-receptor and an essential regulator of planar cell polarity (PCP) and directional cell motility in vertebrate development and embryogenesis [1,2]. In humans, the full-length membrane PTK7 consists of seven extracellular immunoglobulin-like (Ig) domains, a transmembrane region, a juxtamembrane region and a catalytically inert cytoplasmic tyrosine kinase domain [3]. The earlier experimental data suggest that PTK7 directly interacts with β-catenin [4], Wnt proteins [5,6], plexins and semaphorins [7,8], RACK1 and PKCδ1, recruits Dishevelled [9,10] and regulates both the non-canonical Wnt/PCP and canonical Wnt pathways. Because of severe defects in neural tube closure, mice with PTK7 truncation died during embryogenesis [1]. Soluble PTK7 species inhibited angiogenesis by competing with the full-length PTK7 [11].

In contrast to its well-appreciated, crucial role in vertebrate development, the functional importance of the intact full-length PTK7 in malignancy is still a matter of debate. The expression levels of the PTK7 mRNA are highly elevated in colon, gastric, lung cancer and acute myeloid leukemia [12-16]. On the contrary, the levels of PTK7 are decreased in metastatic melanoma [17] and breast carcinoma [18]. Deletions of the chromosome arm 6p, in which the PTK7 gene is localized in humans, were reported in breast cancer [19] and melanoma [20]. Because pericellular proteolysis plays a primary role in cell migration, especially in the directional locomotion of tumor cells, it is likely that proteolysis and PCP converge to promote directed cancer cell migration. In agreement, the functionality of PTK7 is directly regulated by proteolysis. Ubiquitous membrane type-1 matrix metalloproteinase (MT1-MMP), arguably the primary enzyme in pericellular proteolysis and cancer cell migration, cleaves the PKP^621^↓LI sequence in the seventh Ig-like domain of membrane PTK7 and this cleavage results in the liberation of the N-terminal soluble PTK7 fragment (sPTK7) [21]. MT1-MMP proteolysis is followed by cleavage of the PEE↓S^690^ site in the C-terminal residual portion of PTK7 by ADAMs, including ADAM17. The ectodomain shedding by MT1-MMP and ADAMs is a prerequisite for the intramembrane cleavage of PTK7 by γ-secretase. This cleavage releases the C-terminal cytoplasmic tail fragment of PTK7 (ICD), which is then either degraded by the proteasome or transported to the nucleus [11,22].

The limited pre-existing data suggest that the full-length membrane PTK7 and its proteolytic products cause a contrasting effect on the efficiency of cell migration. Thus, the continuing presence of the full-length membrane PTK7 on the plasma membrane down-regulated the myosin light chain (MLC) phosphorylation (a downstream event of the Wnt/PCP pathway) and also reduced migration efficiency of fibrosarcoma HT1080 cells [21]. MT1-MMP proteolysis of PTK7 reversed the inhibitory effect of the full-length membrane PTK7, and resulted in the accumulation of the Stable N-terminal sPTK7 fragment in the extracellular milieu and promoted cell invasion of HT1080 cells [11,21,23]. Expression of the Chz PTK7 mutant that exhibited an additional PEK↓LK^503^ MT1-MMP cleavage site in the junction region between the fifth and the sixth Ig-like domains stimulated cell migration even further [23]. These effects suggest the existence of the intriguing and specific downstream mechanisms by which intact PTK7, its digest fragments and the homo- and heterodimeric complexes between the PTK7 membrane, soluble and intracellular portions control cell function. These mechanisms, however, have not been precisely investigated in the earlier works by us and others [11,21-23]. Understanding these mechanisms will shed additional light on the role that PTK7, alone as well as in combination with MT1-MMP, ADAMs and γ-secretase, plays in cancer cell migration.

In this study, we applied the genome-wide transcriptional and kinome profiling of HT1080 cells to gain insight into the role of the full-length membrane PTK7, and its mutant forms and proteolytic fragments in the downstream signaling and transcriptional events that directly control the efficiency of cell invasion.

## Results

### PTK7 in cell invasion

**Figure 1:**
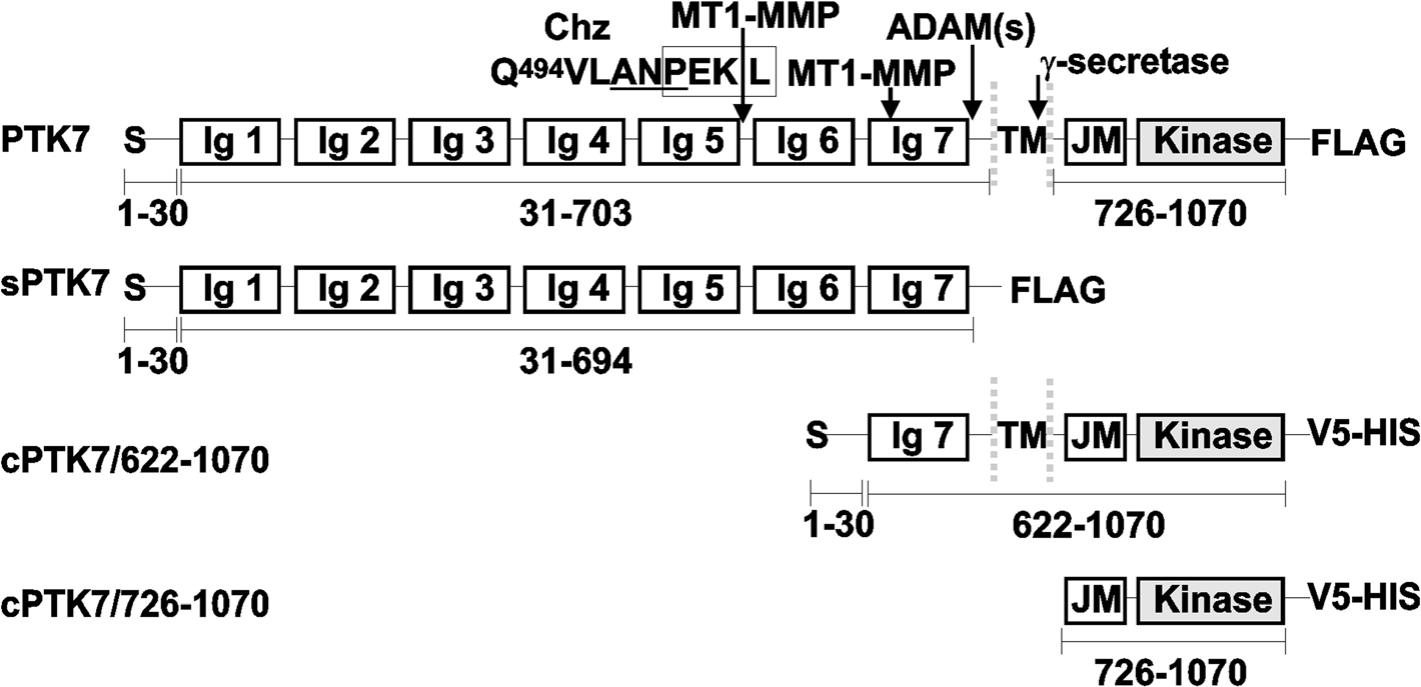
**PTK7 constructs: 1–1070 PTK7, 1–694 sPTK7, cPTK7/622-1070 and cPTK7/726-1070.** S, signal peptide, Ig, immunoglobulin-like domain, TM, transmembrane domain, JM, juxtamembrane region, KINASE, the catalytically inert kinase domain. The amino acid residue numbering is shown below the construct structure. MT1-MMP, ADAM and γ-secretase cleavage sites are indicated by arrows. The Chz mutation (Ala-Asn-Pro, underlined) results in an additional MT1-MMP cleavage site (Pro-Lys-Pro↓Leu^622^; boxed). FLAG and V5-HIS are the C-terminal tags in the recombinant constructs.

To evaluate in detail the effects of PTK7 and its proteolytic fragments on cell invasion, downstream signaling and genome-wide transcriptional regulation, we specifically employed fibrosarcoma HT1080 cells. These highly invasive cells express low endogenous levels of PTK7, but high levels of active MT1-MMP and ADAMs [11]. Because of these parameters, we could manipulate this pseudokinase functionality using HT1080 cells transfected with the recombinant PTK7 constructs. The cells we employed also included the PTK7 knock-out cells (shPTK7 cells), cells with the enforced overexpression of the original membrane PTK7 (PTK7 cells) and the Chuzhoi (Chz) mutant (Chz cells), and, lastly, cells, which overexpressed multiple truncated species of PTK7. These species represented the soluble, the membrane and the cytoplasmic digest fragments that were the result of PTK7 cleavage by MT1-MMP, ADAMs and γ–secretase (sPTK7, cPTK7/622-1070 and cPTK7/726-1070 cells) (Figure 1). In addition, because PTK7 functionality is directly linked to MT1-MMP, we isolated cells in which MT1-MMP was silenced by shRNA (shMT1 cells). We then co-expressed the shMT1 construct with the Chz mutant (shMT1-Chz cells). Expression of these constructs in the same cell system allowed us to determine their downstream effect more precisely.

**Figure 2:**
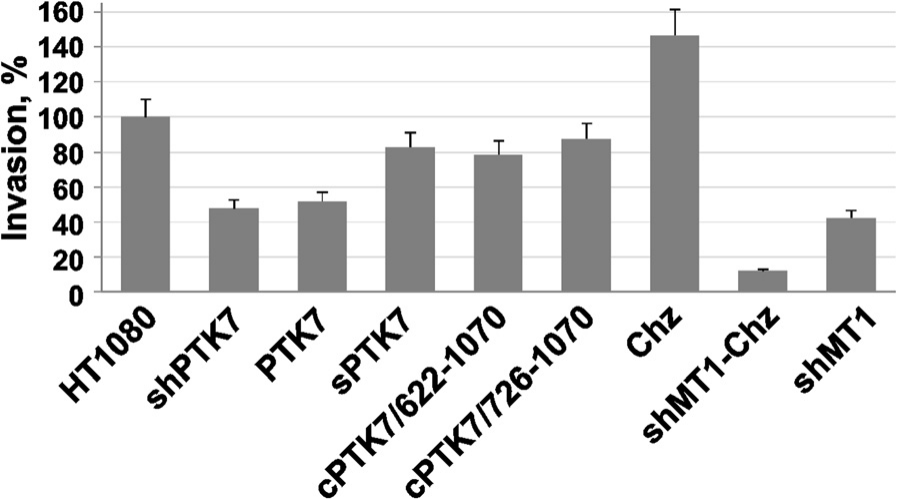
**Cell invasion in collagen type I-coated Transwells.** The levels of cell invasion are shown in percent (*error bars, S.D.*) relative to the HT1080 cell control ( = 100%). FBS (10%) in the lower chamber was used as an attractant. Cells were allowed to invade for 3.5 h. The assays were performed in triplicate.

Our studies revealed that both transcriptional silencing and overexpression of PTK7 inhibited cell invasion by ~50% relative to the parental cells. In turn, Chz stimulated cell invasion by ~50% (Figure 2). Transcriptional silencing of MT1-MMP in shMT1-Chz cells reversed the stimulatory effect of Chz and suppressed cell invasion by ~90%, while MT1-MMP silencing alone (shMT1 cells) was less potent. The effects of sPTK7, cPTK7/622-1070 and cPTK7/726-1070 were less significant.

### Cell microarrays

**Figure 3:**
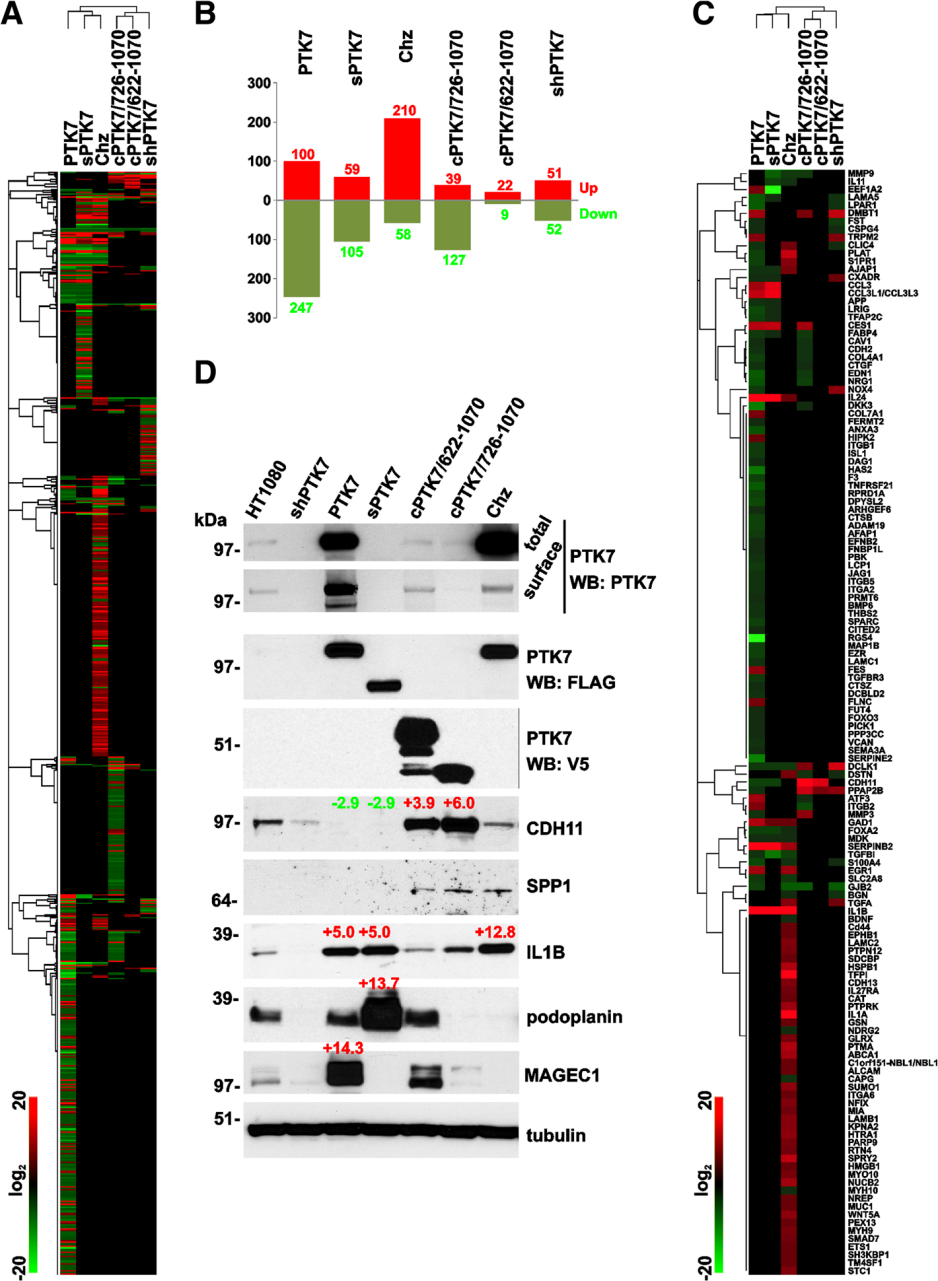
**Genome-wide transcriptional profiling. A**. Heat map and the hierarchical clustering of the expression data (log2 of expression level) of the differentially expressed genes in PTK7, sPTK7, Chz, cPTK7/726-1070, cPTK7/622-1070 and shPTK7 cells relative to the parental HT1080 cells. Red and green correspond to the high and the low expression levels, respectively (color bar inset). **B**. The number of differentially expressed genes in the samples. Numbers show the number of the up-regulated (red) and the down-regulated (green) genes relative to HT1080 cells. **C**. Heat map and the hierarchical clustering of the expression data (log2 of expression level) of the differentially expressed, cell motility-related genes in the cells (selected using the IPA software) relative to HT1080 cells (Additional file 2: Table S2). Red and green correspond to the high and the low expression levels, respectively (color bar inset). **D**. Expression level validation of the selected up-regulated genes by immunoblotting. *Top two panels*, PTK7 was analyzed using the PTK7 antibody in the total cell lysates (total) and the cell surface-biotinylated cell samples (surface). *Bottom panel,* The FLAG- and V5-tagged recombinant PTK7 constructs were analyzed using the FLAG and V5 antibodies (total cell lysate samples), respectively. CDH11, cadherin-11; SPP1, osteopontin; IL1B, interleukin 1β; PDPN, podoplanin; MAGEC1, melanoma antigen family C1. The numbers above the protein bands show the fold-difference of the differentially expressed genes as detected by transcriptional profiling. Tubulin, loading control.

To elucidate the genome-wide effects of PTK7 in its mutant and truncated forms, we performed a whole genome transcriptional analysis of the cells. The differentially expressed genes that exhibited at least a 2-fold difference relative to the parental cells are shown in Additional file 1: Table S1. The hierarchical gene clustering clearly indicated a high similarity among the sPTK7, Chz and PTK7 samples and, on the other hand, among the cPTK7/726-1070, cPTK7/622-1070 and shPTK7 samples (Figure 3A, B). Thus, 67% of the differentially expressed genes overlapped in cPTK7/622-1070 and cPTK7/726-1070 cells. There was a 38.4% overlap between sPTK7 and Chz, and a overlap 44.5% between the sPTK7 and PTK7 samples. The data analysis by the IPA software is described in more detail below.

### shPTK7 cells

shPTK7 cells with PTK7 knock-out exhibited up- and down-regulation of 51 and 52 genes relative to parental HT1080 cells, respectively. The pro-migratory genes were either up-regulated (including DCLK1, DMBT1, FPR1, PPAP2B, NOX4, TRPM2, TGFA, PDE4B and LIN28B) or down-regulated (including S100A4, CSPG4, IL8, LPAR1, BGN, SIRPA and GJB2) in shPTK7 cells (Additional file 2: Table S2). As identified by IPA, Cell-to-Cell Signaling and Interaction, and Cellular Movement were the primary molecular and cellular functions that are likely to be affected in shPTK7 cells.

### PTK7 cells

PTK7 cells with the overexpression of the full-length membrane PTK7 exhibited the up- and down-regulation of 100 and 247 genes relative to HT1080 cells, respectively. 46 (18%) of the down-regulated genes were directly linked to cell migration, including HAS2, LPAR1, TNFRSF21, EDN1, ANXA3, NRG1, S100A4, GJB2, MAP1B, S1PR1, F3, EZR, SPARC, PLAT, CDH11, CTSB, TFAP2C, LCP1, NOX4, DCLK1, ITGB1, FABP4, AFAP1, PPAP2B, CDH2, ITGA2, MMP3, BMP6, PBK, VCAN, ITGB5, THBS2, CTGF, TGFBI, AJAP1, APP, CAV1, CSPG4, ARHGEF6, EFNB2, PRMT6, CTSZ, FNBP1L, MDK, FUT4, and PICK1. In addition, four anti-migratory genes (EEF1A2, GAD1, CES1 and IL24) were up-regulated in PTK7 cells (Figure 3C; Additional file 2: Table S2). Multiple down-regulated pro-migratory genes are directly linked with focal adhesions and cellular protrusions, and organization of the actin cytoskeleton (JAG1, FERMT2, DPYSL2, LCP1, NFIB, EDN1, BMP6, SGK1, PLAT, S1PR1, LAMC, CTGF, CAV1, LPAR1, DCLK1, EZR, CDH2, ITGB1, DAG1, AKAP12, MAP1B, NEFL and NTNG1). Naturally, the most effected molecular and cellular functions predicted by the IPA software analysis were Cellular Movement, Cell Death and Survival, Cellular Assembly and Organization, Cell-to-Cell Signaling and Interaction, and Cellular Development. The affected gene pattern suggested that multiple pathways are likely to be suppressed such as CREBBP, WNT3A, CTNNB1 and TP53. Overall, the data suggest that the regulation of transcriptional activity of the migration-associated genes plays a significant role in the anti-migratory function of the full-length membrane PTK7.

### Chz cells

In Chz cells, 210 and 58 genes were up- and down-regulated relative to HT1080 cells, respectively. In sharp contrast with PTK7 cells, 30 (14%) of the up-regulated genes were pro-migratory (IL1B, IL1A, PLAT, HSPB1, ABCA1, SERPINB2, NUCB2, EGR1, WNT5A, SUMO1, S1PR1, LAMB1, TGFA, KPNA2, MYO10, PTPN12, PARP9, AJAP1, MYH9, MIA, ETS1, NREP, CD44, HMGB1, SDCBP, EPHB1, ITGA6, TM4SF1, GSN, and ALCAM) (Figure 3C; Additional file 2: Table S2). Furthermore, NDRG2 and FOXA2, which are negative regulators of cell motility, were down-regulated. In agreement, Cellular Movement, Cellular Growth and Proliferation, Cell-to-Cell Signaling and Interaction, Cell Cycle, Cell Death and Survival were recognized as the most probable enhanced molecular and Cellular functions in Chz cells. Up-regulation of these cellular functions is likely to involve activation of the IL1B, EGF, RAF1 and down-regulation of the DKK1 pathways.

### sPTK7 cells

59 and 105 genes were up- and down-regulated in sPTK7 cells, respectively. The pro-migratory genes were either up-regulated (including IL1B and CCL3) or down-regulated (including CDH11, MMP9, DCLK1, AJAP1) in sPTK7 cells (Figure 3C; Additional file 2: Table S2). The affected molecular and cellular functions predicted by the IPA were Cell-to-Cell Signaling and Cellular Movement.

### cPTK7/726-1070 cells

These cells expressed the C-terminal 726–1070 membrane fragment that is generated by γ-secretase cleavage of PTK7 and that can be either degraded or transported into the nucleus [11]. 39 and 127 genes were up- and down-regulated in cPTK7/726-1070 cells as compared with HT1080 cells, respectively. The pro-migratory genes were either up-regulated (including CDH11, MMP3 and DCLK1) or down-regulated (including DKK3, CAV1, CDH2, MMP9, ATF3 and DSTN) in cPTK7/726-1070 cells (Figure 3C; Additional file 2: Table S2). The IPA-predicted pathways involved HRAS and KRAS, FGF1, PI3K, MAP2K1, TGFβ, RAC1, MYCN, while Cell Morphology, Cellular Movement, Cell Death and Survival, Cellular Development, Cellular Growth and Proliferation represented the predicted molecular and cells functions.

### cPTK7/622-1070 cells

Only 22 and 9 genes were up- and down-regulated, respectively. As a result, statistically relevant data analysis was not possible in these cells. The top up-regulated gene was cadherin-11 (CDH11).

### Validation of the microarray data by immunoblotting

In our immunoblotting studies, the use of goat and rabbit antibodies against the N- and C-terminal portions of PTK7, respectively, and also the V5 and FLAG antibodies allowed us to discriminate the naturally expressed PTK7 from the recombinant tagged constructs. This analysis confirmed the efficient PTK7 silencing in shPTK7 cells (Figure 3D). Both the naturally expressed and overexpressed PTK7 were efficiently presented at the cell surface in HT1080 and PTK7 cells, respectively. Consistent with our earlier data [23], the cell surface levels of Chz were low because of its efficient shedding by MT1-MMP. Cell surface endogenous PTK7 levels were relatively low in both sPTK7 and cPTK7/726-1070 cells, suggesting that the recombinant constructs affected the cell surface presentation of the full-length membrane pseudokinase. Immunoblotting with the FLAG antibody confirmed that there were the comparable levels of expression of the PTK7, sPTK7 and Chz constructs in the respective cells. Immunoblotting with the V5 antibody demonstrated the proteolysis of the cPTK7/622-1070 construct. As a result of this proteolysis, the cPTK7/622-1070 construct was partially converted into the γ-secretase-dependent species similar to cPTK7/726-1070.

We then employed immunoblotting to validate the levels of the selected genes, up- and down-regulation of which was detected by the genome-wide transcriptional profiling of the respective cells. Thus, the cadherin-11 (CDH11) gene expression was up-regulated 3.9- and 6-fold in cPTK7/622-1070 and cPTK7/726-1070 cells, respectively, and down-regulated 2.9-fold in both PTK7 and sPTK7 cells. In agreement, cadherin-11 protein was significantly elevated in cPTK7/622-1070 and cPTK7/726-1070 cells, and decreased in PTK7 and sPTK7 cells. Cadherin-11 plays an important role in osteogenesis, epithelial-to-mesenchymal transition and bone metastasis [24-26], and is functionally linked to osteopontin (SPP1), a bone-specific protein. In agreement, the levels of osteopontin increased in cPTK7/622-1070, cPTK7/726-1070 and Chz cells relative to other cell types.

According to the microarray data, IL1B was up-regulated 12.8-, 5- and 5-fold in Chz, PTK7 and sPTK7 cells, respectively. In agreement, immunoblotting recorded a significant IL1B increase in PTK7, sPTK7 and Chz cells. An inflammatory IL1B cytokine is involved in cancer onset and progression [27,28].

Our microarray results suggested that podoplanin (PDPN) was up-regulated 13-fold in sPTK7 cells. In agreement, our immunoblotting studies revealed that the PDPN expression was greatly elevated in sPTK7 compared with other cell types. PDPN, a mucin-type transmembrane protein, is important to embryo development and epithelial-to-mesenchymal transition. PDPN is frequently upregulated in metastatic cancers to induce RhoA activity and promote cell migration and invasion [29-31]. In agreement, our previous data suggest that RhoA is significantly activated in sPTK7 cells, potentially, *via* the PDPN-dependent mechanism [21].

As the microarray data demonstrated, MAGEC1 (melanoma antigen family C1) expression was up-regulated 14-fold in PTK7 cells. Consistently, the MAGEC1 levels were elevated in PTK7 cells. MAGEC1 belongs to cancer/testis (CT) antigen family. The expression of MAGEC1 is frequently elevated in a variety of cancers [32,33].

### Phospho-kinase array

To get a deeper insight into the PTK7-dependent regulation of cell signaling, we employed the Proteome Profiler Human Phospho-Kinase Array. The use of this array permits a comparative analysis of 43 kinase phosphorylation sites.

**Figure 4:**
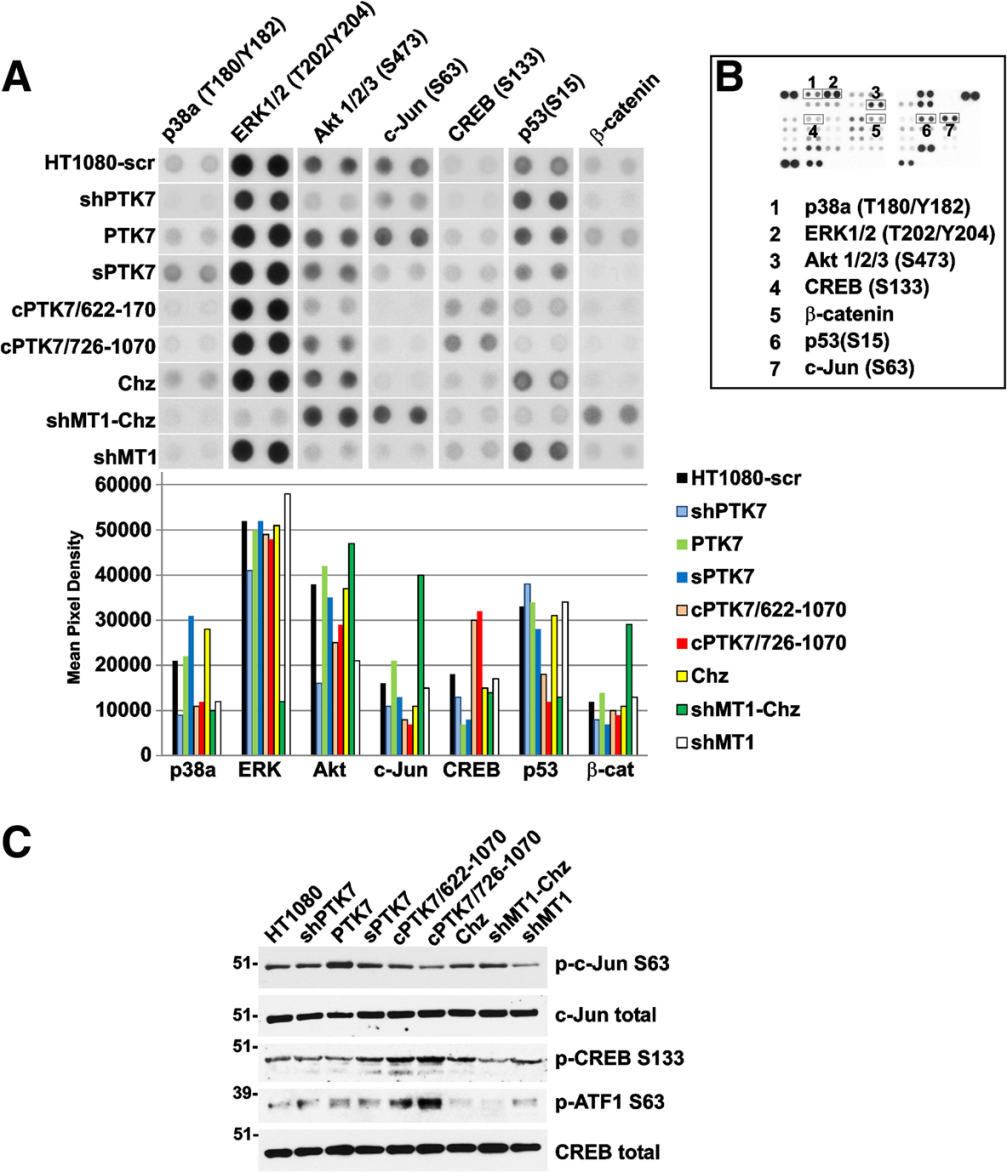
**Protein phosphorylation profiling. A**. *Top panel,* Protein phosphorylation profiling of p38a (T180/Y182), ERK1/2 (T202/Y204), Akt 1/2/3 (S473), CREB (S133), β-catenin protein, p53 (S15) and c-Jun (S63) in HT1080-scr, shPTK7, PTK7, sPTK7, cPTK7/622-1070, cPTK7/726-1070, Chz, shMT1-Chz and shMT1 cells. The spots were scanned and digitized. Pixel density of the spots is shown by the graphs in the bottom panel. **B**. A representative array of the HT1080 cell sample. The spots (boxed) of p38a (T180/Y182), ERK1/2 (T202/Y204), Akt 1/2/3 (S473), CREB (S133), β-catenin protein, p53 (S15) and c-Jun (S63) are numbered. **C**. Western blotting of phosphorylated and total cell c-Jun, CREB and ATF1 in HT1080, shPTK7, PTK7, sPTK7, cPTK7/622-1070, cPTK7/726-1070, Chz, shMT1-Chz and shMT1 cells.

Our array data suggested that the PTK7 transcriptional silencing decreased phosphorylation of p38a (T180/Y182), ERK1/2 (T202/Y204, T185/Y187), Akt (S473, T308), c-Jun (S63) and CREB (S133) in shPTK7 cells and increased phosphorylation of p53 (S15) relative to the HT1080 cell control transfected with the scrambled shRNA construct (Figure 4). The levels of β-catenin were also decreased. A decrease in phosphorylation of c-Jun (S63) and in the β-catenin protein levels were recorded in sPTK7 cells.

A significant increase in phosphorylation of CREB (S133) and a decrease in p38a (T180/Y182) and c-Jun (S63) were recorded in cPTK7/726-1070 cells. In a way that is similar with cPTK7/726-1070 cells, enhanced and repressed phosphorylation of CREB (S133) and c-Jun (S63) were the characteristic features of cPTK7/622-1070 cells, respectively. In shMT1-Chz cells, the most evident was the reduced phosphorylation of ERK1/2 (T202/Y204, T185/Y187) and p53 (S15) relative to Chz and shMT1 cells. On the contrary, the levels of phosphorylated Akt (S473, T308) and c-Jun (S63) and of the total β-catenin protein increased in shMT1-Chz cells.

**Figure 5:**
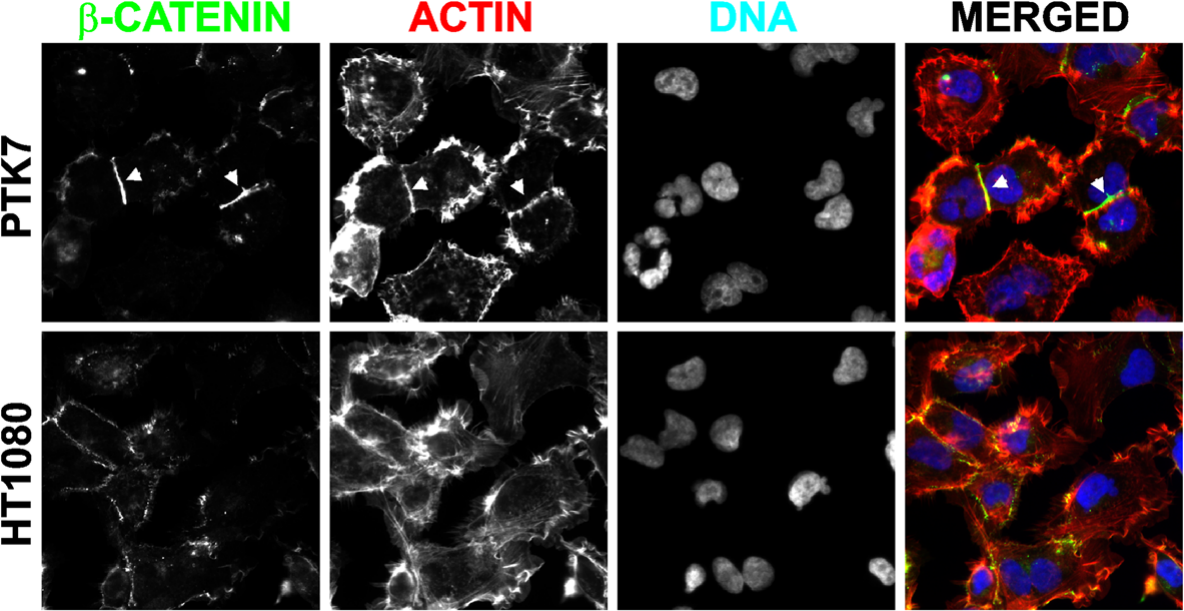
**Immunostaining of β-catenin in HT1080 and PTK7 cells.** Cells were stained using the β-catenin monoclonal antibody. The actin cytoskeleton was stained using Alexa Fluor 594-conjugated phalloidin. DAPI, the nuclei. Arrows indicate the β-catenin immunoreactivity accumulation in the cell-to-cell contacts in PTK7 cells.

To corroborate the kinome array data, we analyzed the levels of p-c-Jun (S63) and p-CREB (S133) in the total cell lysate of HT1080, shPTK7, PTK7, sPTK7, cPTK7/622-1070, cPTK7/726-1070, Chz, shMT1-Chz and shMT1 cells (Figure 4C). We also determined levels of ATF1, a transcription factor closely related to CREB. In agreement with the kinome array data, p-c-Jun phosphorylation was enhanced in PTK7 cells as compared with HT1080 cells, and also in shMT1-Chz cells relative to shMT1 cells. In addition, we recorded an increase in p-CREB (S133) and p-ATF1 (S63) in cPTK7/622-1070 and cPTK7/726-1070 cells as compared with other cells. To further support the increased levels of β-catenin we observed in the kinome array in PTK7 cells, we performed immunostaining of HT1080 and PTK7 cells. As expected, the increased β-catenin immunoreactivity at the cell-to-cell contacts was readily observed in PTK7 cells but not in HT1080 cells (Figure 5).

**Figure 6:**
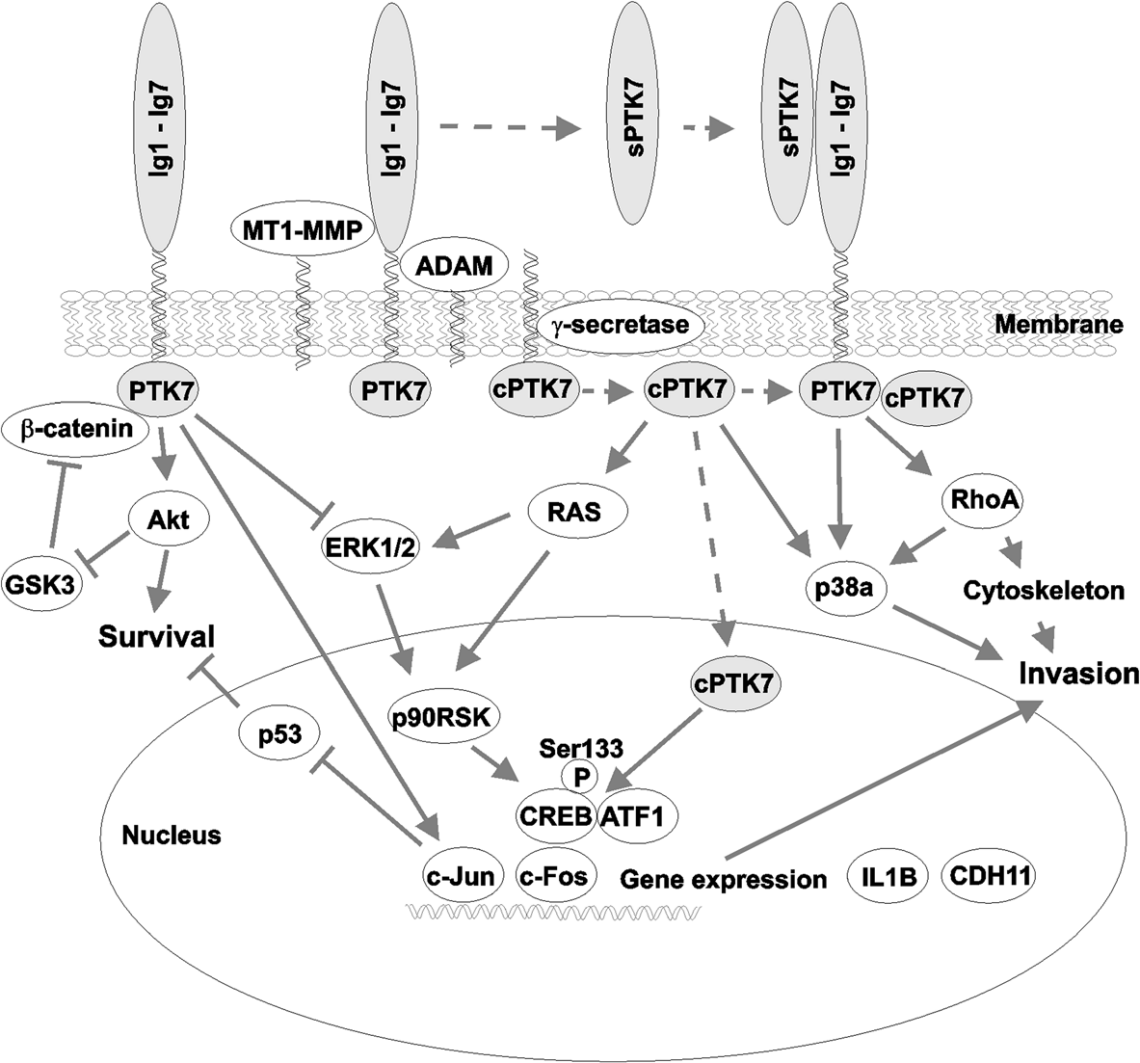
**PTK7 signaling.** The pathways and the key proteins are predicted based on the results of our genome-wide transcriptional and protein phosphorylation profiling. Our data suggest that the stabilized full-length membrane PTK7 up-regulates the Akt and c-Jun pathways, and down-regulates the p53 pathways leading to the cell survival signaling. Full-length PTK7 also suppresses ERK and CREB-mediated gene expression and cell motility. The proteolysis of PTK7 ectodomain (Ig1-Ig7) by MT1-MMP and ADAMs generates the soluble sPTK7 form. The subsequent cleavage of the C-terminal membrane PTK7 fragment by γ-secretase releases the intracellular domain (cPTK7). The soluble sPTK7 species binds the full-length membrane PTK7 and activates the RhoA [21] and p38a pathways, which regulate the actin cytoskeleton and the expression of the migratory genes, including IL1B. The intracellular PTK7 domain fragment, cPTK7, enters the cell nucleus and induces the RAS/p90RSK/CREB signaling resulting in the CREB and ATF1 phosphorylation at Ser133 and Ser63, respectively, and the expression of the pro-migratory genes, including cadherin-11 (CDH11). We hypothesize that the ratio of the full-length membrane PTK7 to the N-terminal and the C-terminal PTK7 proteolytic fragments acts as a switch, which establishes the downstream signaling pathways that control cell survival and motility.

The data, especially when combined, led us to conclude that the N-terminal ectodomain and the C-terminal cytoplasmic portions differentially regulate multiple signaling pathways. The summary of our data and the predicted regulatory signaling pathways are presented in Figure 6.

## Discussion

Pseudokinase PTK7, a functionally important regulator of Wnt pathways [1-6], is a subject of the multi-step proteolysis in multiple cell/tissue types [11,21-23]. PTK7 levels need to be tightly controlled to enable migration of HT1080 cancer cells. The full-length membrane PTK7 has a pronounced anti-migratory effect in HT1080 cells while its proteolysis reverses this anti-migratory effect and facilitates cell locomotion. The recent data suggest that proteolysis of the full-length membrane PTK7 involves MT1-MMP, ADAMs and γ-secretase [11,21-23]. The resulting multiple and stable digest fragments of PTK7 may be either liberated into the extracellular milieu or retained on the plasma membrane or released into the cytoplasm and then transported into the nucleus. We hypothesized that certain PTK7 fragments display a distinct, albeit currently unknown, function relative to intact PTK7. To test this hypothesis, we employed fibrosarcoma HT1080 cells, which expressed the full-length membrane PTK7 or its truncated and mutant forms that corresponded to the PTK7 digest fragments. We also used the cells in which PTK7 and MT1-MMP were transcriptionally silenced. The analysis of PTK7 expression suggests that the PTK7 proteolytic fragments modulate the full-length PTK7 cell surface presentation and function.

We demonstrated that MT1-MMP silencing in shMT1-Chz cells inhibited proteolytic processing of the Chz mutant and both induced accumulation of the full-length Chz at the cell surface and markedly reduced cell invasion [11]. Thus, we used the shMT1-Chz cells to reveal the signaling by the stabilized full-length PTK7. Based on the phospho-kinase array analysis of shMT1-Chz cells, we now suggest that the stabilized full-length PTK7 up-regulates Akt and c-Jun signaling. It is well documented that Akt regulates cellular survival pathways, inhibits GSK3 and modulates Wnt signaling [34,35]. c-Jun is a proto-oncogene that represses p53 transcription, protects cells from apoptosis and induces cell cycle progression [36]. In agreement, the levels of p53 (S15) were reduced while the levels of β-catenin were elevated in shMT1-Chz cells. It was also demonstrated by others that PTK7 silencing induced apoptosis in colon cancer HCT-116 cells, reinforcing that PTK7 regulates cell survival [37].

Furthermore, our analysis of the signaling pathways predicted that WNT3A and CTNNB1 (β-catenin) were inhibited in PTK7 cells, suggesting that β-catenin was stabilized by PTK7 and was not efficiently translocated into the cell nucleus. The recent study that documented direct interactions between PTK7 and β-catenin supports our data [4]. In agreement, our results indicated an accumulation of the β-catenin immunoreactivity at the cell-to-cell junctions in PTK7 cells. Interestingly, the sPTK7 construct reduced both the level of phosphorylated c-Jun (S63) and the β-catenin level, suggesting an antagonistic role of sPTK7 relative to the full-length PTK7. We hypothesize that the c-Jun activity is specifically induced by the intact full-length PTK7. On the contrary, both C-terminal PTK7 forms reduced the levels of c-Jun (S63) and induced phosphorylation of CREB (S133) and ATF1 (S63), a transcription factor similar to CREB. Phosphorylation at Ser133 activates the CREB transcriptional activity [38,39]. In agreement, our IPA analysis of the differentially expressed genes in cPTK7/726-1070 cells predicted the activation of HRAS, KRAS and FGF1, the upstream pathways that control CREB activity [40]. Overall, it is likely that MT1-MMP proteolysis of PTK7, ADAM and γ-secretase promotes cancer progression by stimulating the CREB activity. Notably, CREB activity is elevated in metastatic cancers [41,42] and essential to embryo development and neurogenesis [43]. It is tempting to hypothesize that the activation of CREB by the C-terminal PTK7 fragments contributes to both normal embryogenesis and cancer. CREB activity is also regulated by CREB-binding protein (CBP or CREBBP). Consistently, our gene array analysis predicted the down-regulation of the CREBBP pathway in PTK7 cells, suggesting further that the CREB pathway is differentially regulated by the full-length PTK7 and the C-terminal PTK7 fragments.

In addition, the C-terminal PTK7 species up-regulated cadherin-11 (also called OB-cadherin), a calcium-dependent cell-cell adhesion molecule and a mesenchymal cell marker [24]. Cadherin-11 is expressed in the mesoderm-derived tissues, associated with the epithelial-to-mesenchymal transition, and regulates osteogenesis [25] and cancer metastasis to the bone [26]. Cadherin-11 is also involved in cell migration in embryogenesis [24,25,32]. It is likely that PTK7 and cadherin-11 interact in embryogenesis and regulate similar developmental processes. Furthermore, PTK7 and cadherin-11 are predicted to be co-expressed by the CoexpressDB open source software.

## Conclusions

Overall, by using genome-wide transcriptional and phospho-kinase profiling of the cell samples, we determined that the full-length membrane PTK7 and its N-terminal and C-terminal proteolytic fragments differentially regulate multiple genes and proteins involved in cell motility. These differences were most evident in the phosphorylation levels of p38a, ERK, Akt, c-Jun, CREB, ATF1, p53, and in the β-catenin and cadherin-11 protein levels. Our data correlate well with the results in other receptor tyrosine kinases and support the general idea about the distinct functions of the full-length receptor and its intracellular domain (ICD) fragment [44]. The results generated from our model system will shed more light on the PTK7 functionality in both physiological and pathological conditions.

## Methods

### Antibodies and reagents

General reagents were purchased from Sigma-Aldrich (Saint Louis, MO, USA). A goat polyclonal AF4499 antibody against the N-terminal 31–199 portion of PTK7, a goat polyclonal antibody to IL1B and a rabbit polyclonal antibody to pCREB (S133) were from R&D Systems (Minneapolis, MN, USA). A mouse monoclonal antibody to the V5 tag was from Invitrogen (Carlsbad, CA, USA) A mouse monoclonal M2 antibody to the FLAG tag was from Sigma-Aldrich. The rabbit polyclonal antibodies to cadherin-11 (CDH11, OB-cadherin) and α-tubulin, rabbit monoclonal antibodies to CREB, podoplanin and p-c-Jun (S63), and a mouse monoclonal antibody to c-Jun were from Cell Signaling Technology (Danvers, MA, USA). A rabbit monoclonal antibody to p-ATF1 (S63) was from Abcam (Cambridge, MA, USA). The mouse monoclonal antibodies to osteopontin (SPP1) and MAGEC1 were from EMD Millipore (Temecula, CA, USA) and Santa Cruz Biotechnology (Dallas, TX, USA), respectively. The species-specific HRP-conjugated secondary antibodies were from Fitzgerald Industries (Acton, MA, USA).

### Cells, cloning and mutagenesis

Human fibrosarcoma HT1080 cells (HT1080 cells) were from ATCC (Manassas, VA, USA). HT1080 cells transfected with the full-length 1–1070 PTK7 containing the C-terminal FLAG tag (PTK7 cells), the N-terminal 1–694 fragment with the C-terminal FLAG tag (sPTK7 cells) and the C-terminal 726–1070 fragment with the V5-HIS tag (cPTK7/726-1070 cells) were described earlier [11,21,23]. Cells in which the transcription of the MT1-MMP gene was silenced using the shRNA construct (shMT1 cells) were characterized earlier [11,45]. The C-terminal 622–1070 membrane fragment (cPTK7/622-1070 cells) that corresponded to the MT1-MMP cleavage fragment of the full-length membrane PTK7 was generated by PCR using the full-length wild-type PTK7-1 cDNA (OriGene, Rockville, MD, USA) as a template, and 5′-GGTACCCAGACAGCCCTGATTCAGTGGAAAGG-3′ and 5′-CGGCTTGCTGTCCAC-3′ as the forward and reverse primers, respectively. The 1–30 PTK7 signal peptide sequence was amplified using 5′-CACCATGGGAGCTGCGCGGGGATC-3′ and 5′-CCTTTCCACTGAATCAGGGCTGTCTGGGTACC-3′ as the forward and reverse primers, respectively, and then the signal peptide sequence was inserted at the N-terminus to ensure delivery of the cPTK7/622-1070 construct to the plasma membrane. The resulting secretory cPTK7/622-1070 construct was sub-cloned into the pcDNA3.1D/V5-His-TOPO vector (Invitrogen) and then used to stably transfect HT1080 cells using Lipofectamine LTX (Invitrogen). Stably transfected cells were selected in the presence of G418 (200 μg/ml). Cell expression of the cPTK7/622-1070 construct was confirmed using Western blotting with the V5 antibody.

The 29-mer shRNA constructs in the retroviral RFP vector (pRFP-C-RS) (Origene; catalog number TF200451) were used to silence the endogenous PTK7 in HT1080 cells. Following Lipofectamine LTX transfection of the cells with the vector, the RFP-positive cells were selected using a cell sorter. Selected cells were then grown in the presence of puromycin (2 μg/ml). Immunoblotting with the goat polyclonal PTK7 antibody was used to select the silenced clones. Three selected clones with the silenced PTK7 were pooled together (shPTK7 cells). Similarly, the scrambled shRNA constructs were used to generate the HT1080-scr cell control.

### Invasion assay

The invasion assay was performed in triplicate in wells of a 24-well Transwell plate with an 8-μm pore size membrane [11]. The membranes of the Transwell inserts were coated with type I collagen (30 μg/well, BD Biosciences, San Jose, CA, USA). Cells (1 × 10^5^/well) in serum-free DMEM (0.1 ml) were placed into the upper chamber. The 10% fetal bovine serum (FBS)-containing DMEM (used as a chemoattractant, 0.6 ml) was placed in the lower chamber. Serum-free DMEM (0.6 ml) was used as a control. Cells were allowed to invade for 3.5 h. The cells were stained for 10 min with 0.2% crystal violet/20% methanol (0.3 ml). The cells on the upper membrane surface were removed with a cotton swab. The dye from the cells that migrated onto the lower surface of the membrane was extracted with 1% SDS (0.25 ml). The resulting A570 nm was measured using a plate reader.

### Genome-wide transcriptional profiling and data analysis

Cells (1 × 10^4^/ml) were plated in DMEM-10% FBS in a 100-mm dish and grown for 72 h to produce a subconfluent culture. Total cellular RNA was extracted using a Direct-zol RNA MiniPrep kit (Zymo Research, Irvine, CA, USA). Biotin-labeled cRNA samples were prepared using an RNA Amplification Kit (Life Technologies, Grand Island, NY, USA). The labeled cRNA (750 ng) was hybridized for 18 h at 58°C to the HumanHT-12 v4 Expression BeadChip with over 46,000 gene transcripts (Illumina, San Diego, CA, USA). BeadChips were then developed using fluorolink streptavidin-Cy3 (GE Healthcare, Piscataway, NJ, USA). Array chips were scanned using an Illumina BeadArray Reader. The initial data extraction and normalization were performed using the BeadArray Reader and GeneSpring GX software (Agilent, Santa Clara, CA, USA). The differentially expressed genes (compared to parental HT1080 cells) were identified based on the Welch’s t test and -fold difference of the expression level (cutoff >2-fold difference, p-value <0.05). Heatmaps and hierarchical clustering were generated using the GenePattern open-source software package (Broad Institute, Cambridge, MA, USA). The pathway and functional analyses were conducted using the Ingenuity Pathway Analysis (IPA) software (Ingenuity Systems, Redwood City, CA, USA). The microarray data were deposited to the Gene Expression Omnibus (GEO) data base with the accession number GSE53340.

### Human phospho-kinase array

The phosphorylation profile was analyzed in the cells using the Proteome Profiler Human Phospho-Kinase Array (R&D Systems). Cells (1 × 10^4^/ml) were plated in DMAM-10% FBS in a 100-mm dish and grown for 72 h to produce a subconfluent culture. Cell lysate samples (0.5 mg; 1.5 mg/ml each) were applied per array set comprised of two nitrocellulose membranes with the spotted capture antibodies. The bound material was detected using the biotinylated antibodies followed by streptavidin conjugated with horseradish peroxidase. The chemiluminescent signal was acquired using the HyBlot CL autoradiography film (Denville, South Plainfield, NJ, USA). The film was scanned and digitized. Pixel density of the spots was quantified using the ImageJ software.

### Cell surface biotinylation and total cell lysates

Cell surface proteins were biotinylated by incubating cells for 1 h on ice in PBS containing 0.1 mg/ml EZ-Link sulfosuccinimidyl 2-(biotinamido)-ethyl-1,3-dithiopropionate (Thermo Fisher Scientific, Rockford, IL, USA). Cells were lysed in 20 mM Tris–HCl buffer, pH 7.4, containing 150 mM NaCl, 1% deoxycholate, 1% octylphenoxypolyethoxyethanol (IGEPAL), protease inhibitor mixture set III, 1 mM phenylmethylsulfonyl fluoride and 10 mM EDTA. Biotinylated proteins were then captured using streptavidin-agarose beads. Biotinylated proteins were eluted from the beads using 2X SDS sample loading buffer (125 mM Tris–HCl, pH 6.8, containing 4% SDS, 0.005% bromophenol blue, 20% glycerol and 20 mM DTT).

For preparation of the total cell lysate samples, cells were lysed using the cell lysis buffer of the Proteome Profiler Human Phospho-Kinase Array kit (R&D Systems). The samples were centrifuged at 4°C (14,000 rpm, 15 min). The protein concentration in the supernatant samples was adjusted to 1.5 mg/ml. The sample aliquots (30 μg total protein each) were separated by SDS-gel electrophoresis in the 4-12% gels and analyzed by immunoblotting with the specific primary antibodies followed by the horseradish peroxidase-conjugated species-specific secondary antibody and the SuperSignal West Dura extended duration substrate (Thermo Fisher Scientific). The chemiluminescent signal was acquired using the HyBlot CL autoradiography film.

### Cell immunostaining

Cells grown on a microscope coverglass (Thermo Fisher Scientific) were fixed using 4% *p*-formaldehyde, permeabilized using 0.1% Triton X-100, and blocked in 1% casein. Cells were stained using the primary monoclonal mouse antibody to β-catenin (1:1,000 dilution) (Becton Dickinson, Franklin Lakes, NJ, USA) for 16 h at 4°C followed by Alexa Fluor 488-conjugated anti-mouse secondary antibody (1:500 dilution). The Alexa Fluor 594-conjugated phalloidin (1:500 dilution) (Thermo Fisher Scientific) was used to visualize the actin cytoskeleton. The specimens were mounted in the Vectashield mounting medium with 4′,6-diamidino-2-phenylindole (DAPI) (Vector Laboratories, Burlingame, CA, USA). Images were acquired using an Olympus BX51 fluorescence microscope equipped with a MagnaFire digital camera and MagnaFire 2.1C software (Olympus, Center Valley, PA, USA).

## Abbreviations

ADAM: A disintegrin and metalloprotease; Chz: Chuzhoi mutant of PTK7; ICD: Intracellular domain; MT1-MMP: Membrane type-1 matrix metalloproteinase; PCP: Planar cell polarity; PTK7: Protein tyrosine kinase 7; cPTK7/622-1070 and cPTK7/726-1070: The C-terminal, cytoplasmic 622–1070 and 726–1070 fragments of PTK7; Respectively; sPTK7: The N-terminal soluble PTK7 fragment; shMT1: MT1-MMP knockout; shPTK7: PTK7 knockout.

## Competing interests

Authors declare that there are no competing interests.

## Authors’ contributions

VSG carried out the studies. VSG and AYS wrote the manuscript. All authors read and approved the final manuscript.

## Supplementary Material

Additional file 1: Table S1 Genome-wide transcriptional profiling of Chz, shPTK7, cPTK7/622-1070, cPTK7/726-1070, sPTK7 and PTK7 cells relative to the parental fibrosarcoma HTR1080 cells. Click here for file

Additional file 2: Table S2 Genome-wide transcriptional profiling of the cell migration-related genes in Chz, shPTK7, cPTK7/622-1070, cPTK7/726-1070, sPTK7 and PTK7 cells relative to the parental fibrosarcoma HTR1080 cells. Click here for file
